# Report of *Tuckerella pavoniformis* (Acari: Tuckerellidae) on Mamey, *Mammea americana* (Calophyllaceae), in Northwestern Peru

**DOI:** 10.3390/insects13050473

**Published:** 2022-05-18

**Authors:** Hector Alonso Escobar-Garcia, Jennifer J. Beard, Ronald Ochoa

**Affiliations:** 1 Facultad de Agronomía, Universidad Nacional de Piura (UNP), Piura 20002, Peru; 2 Estación Experimental Agraria Los Cedros, Instituto Nacional de Innovación Agraria (INIA), Tumbes 24500, Peru; 3 Queensland Museum, South Brisbane, QLD 4101, Australia; jenny.beard@qm.qld.gov.au; 4 Systematic Entomology Laboratory, United States Department of Agriculture, Agricultural Research Service, Beltsville, MD 20705, USA; ron.ochoa@usda.gov

**Keywords:** agroforestry system, exotic fruit, emerging pest, peacock mites, Tetranychoidea

## Abstract

**Simple Summary:**

*Tuckerella pavoniformis* (Ewing) (Acari: Tuckerellidae) was found to be solidly associated with the tropical fruit mamey, *Mammea americana* L. (Calophyllaceae), for the first time in northwestern Peru. The highest *T. pavoniformis* population density was located on the epicarps of fruits. Biometric data was collected from mite-infested fruit for future comparisons with mite-free fruit. The localized commercialization of this fruit could play an important role in the spread of this mite within Peru.

**Abstract:**

The family Tuckerellidae, or peacock mites, is a monogeneric group comprising approximately 32 species, which are usually collected from the fruits or woody parts of their host plants. Fruits and branchlets of mamey, *Mammea americana* L. (Calophyllaceae) trees in north-western Peru were sampled for peacock mites throughout spring and summer for two consecutive years. This is the first record of *Tuckerella pavoniformis* (Ewing) (Acari: Tuckerellidae) feeding on mamey. Aggregations of mites were much higher and more common on the fruit epicarps than on branchlets. Recommendations for the development of an Integrated Pest Management strategy for this peacock mite are included.

## 1. Introduction

*Tuckerella pavoniformis* (Ewing) (Acari: Tetranychoidea: Tuckerellidae), the first peacock mite ever recorded, was originally described in 1922 as *Eupalopsis pavoniformis*. The description was based on material collected on *Hibiscus sp*. (Malvaceae) imported into San Francisco from Hawaii, USA [1]. Shortly after that, *Tuckerella ornata* (Tucker) was described as *Tenuipalpus ornatus* in the family Tetranychidae from South Africa in 1926, based on material collected from oranges [2]. Womersley [3] erected the new genus *Tuckerella* for *T. ornata*, keeping it within the Tetranychidae. In 1953, Baker and Pritchard [4] evaluated and updated the superfamily Tetranychoidea, and in doing so proposed the new family Tuckerellidae to accommodate the only two species known at that time, *T. ornata* and *T. pavoniformis*, and included host plant records for both species. According to Baker and Pritchard [4], *T. pavoniformis* had been recorded from *Hibiscus* sp. and papaya fruit (Caricaceae) in Hawaii; and from *Citrus* sp. (Rutaceae), cypress pine (*Callitris* sp., Cupressaceae), *Eucalyptus* sp. (Myrtaceae), and privet (*Ligustrum* sp., Oleaceae) in Australia. However, we now know that the Australian mite species records were based on misidentifications made by Womersley [3], and are known to actually represent *T. flabellifera* Miller [5]. Baker and Pritchard [4] also provided a host list for *T. ornata*, which included the tropical fruit called mamey or mamey apple, *Mammea americana* L. (Calophyllaceae) which had been imported into San Diego, USA, from Guatemala. Additionally, Ochoa et al. [6] reported *T. pavoniformis* damaging the fruit of *Psidium cattleianum* Sabine (Myrtaceae) in Central America. 

*Mammea americana* is native to the West Indies and northern South America. The tree is grown in small-scale local commercial plantations in various Central and South American countries for beverage manufacture, and the edible pulp [7] which is exported to various countries around the world. This fruit tree is frequently used in border plantings and windbreaks for other commercial plantations (such as banana, cocoa and mango), and is domestically grown as an ornamental or backyard tree [8,9,10]. *Mammea americana* is known to have many medicinal, and pharmaceutical uses, which include insect-repellent properties [8,11,12]. Additionally, endophytic fungi of *M. americana* have been identified as a source of active secondary metabolites with non-toxic antimicrobial properties [13]. Interestingly, even the shell of the fruit is useful as an effective bioadsorbent of chrome [14]. In Singucate, Piura, Peru, mamey is grown in cocoa plantations within agroforestry systems.

This tropical tree has dark green, shiny, leathery, elliptical leaves. The creamy white, fragrant flowers [15], are borne singly or in clusters of two or three on short stalks in the axils of young branches [16]. Recent studies indicate that this species exhibits a “cryptic″ androdioecy in that there are individuals with male flowers and individuals with functionally female hermaphroditic flowers [17]. Young mamey trees begin to produce flowers and fruits between eight and 13 years of age, and fruit production is then consistent from year to year [18]. The fruit is a large globose berry [19,20] attached to the plant by a short, thick stem, and takes more than a year to mature [21]. The epicarp is grayish brown, verrucose, and rough to the touch due to small latex globules of variable thickness and consistency; and it remains hard until fully mature at which point it softens slightly. The edible endocarp, is light yellow to golden orange, non-fibrous, and may have one to four seeds [9,15]. At maturation, the endocarp separates from the rest of the pericarp and testa [19].

Mamey fruits are marketed nationally in Peru and the main productive regions are Tumbes, Piura, Lambayeque, La Libertad and Cajamarca. In 2020, Peru exported 27,548 kg of mamey pulp to Europe, mostly to Spain, Netherlands, France, Germany, and Italy [22]. This article reports *M. americana* as a new host tree association for *T. pavoniformis* in Peru, with observations on the ecology of the mite and mamey trees, and offers contributions to the development of Integrated Pest Management strategies.

## 2. Materials and Methods

### 2.1. Studied Area

**Figure 1:**
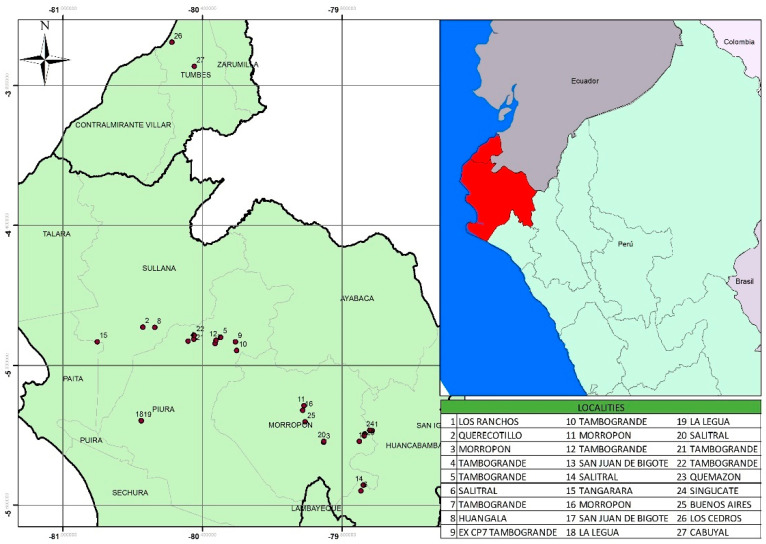
Current peacock mite *Tuckerella pavoniformis* distribution in north–western Peru.

In this study, *T. pavoniformis* mites were collected in agricultural plots in 27 localities of two Departments, Tumbes and Piura (Figure 1), located in north–western Peru. The fruits were harvested in the years 2020 and 2021 during the period of fruit maturity that coincides with spring and summer. The climate in Tumbes and Piura is considered to be tropical and subtropical desert, or a BWh type according to the Köppen–Geiger climate classification system. 

**Figure 2:**
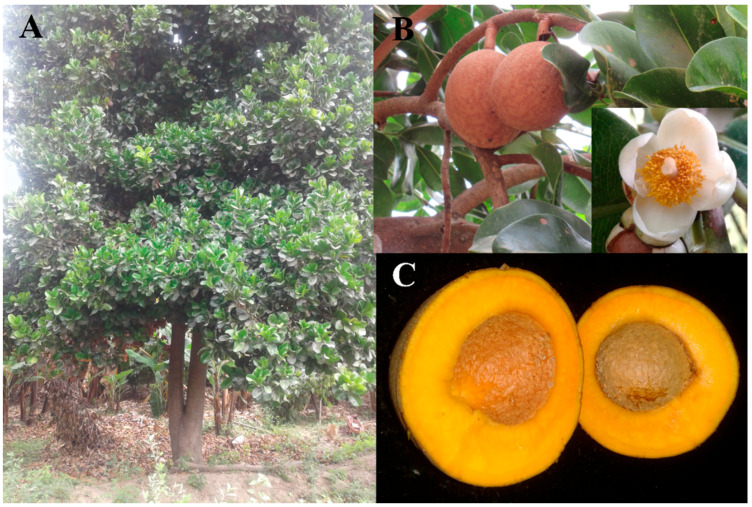
(**A**) Adult tree of *Mammea americana* L. (Calophyllaceae). (**B**) Detail of the fruits and leaves of mamey, with insertion of detail of the flower. (**C**) Cross sections of halved mamey apple fruit, *M. americana*.

**Figure 3:**
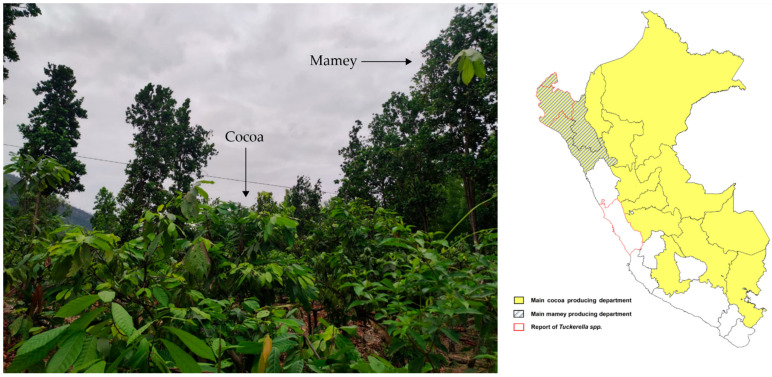
Current *Tuckerella* spp. distribution in Perú on mamey and cocoa crops.

The sampled mamey trees (Figure 2) were between 15 and 20 m tall, 20 to 25 years old, and individual trees were numbered from one to ten within each agricultural plot surveyed. The one exception was the town of Singucate where mamey is used in cocoa cultivation as part of an agroforestry system (Figure 3). A single tree was surveyed within each plot, when multiple trees were present one was randomly selected. Each mamey fruit takes more than a year to mature, and the timing of maturation in the surveyed localities occurred between October and February. Local management practices for mamey trees in the surveyed region included selective pruning of dry branches, irrigation, and manual weed control every month. The trees were pesticide free because tree height made it impractical to spray.

### 2.2. Peacock Mite Sampling and Identification

Selected mamey trees in 27 separate plots were sampled for peacock mites (Figure 1). Each sample consisted of two fully developed fruit, and two 15 cm long branchlets taken from the lower third of one tree. Fruit and branchlets were put in separate paper bags, transported to the laboratory inside a portable cooler, and examined under a stereomicroscope (ZEISS Stemi 508 with a digital camera AixocamERc 5s). The numbers of mites on a randomly chosen 1 cm^2^ area of fruit epicarp, and on the entire branchlets, were recorded. The mites were stored in 2 mL microtubes with 70% alcohol. After one month, were mounted in Hoyer’s medium [23], and stored in an oven at 50 °C for 5 days. Mites were identified using taxonomic keys provided by Meyer and Ueckermann [24], and their most important taxonomic characters, using an Omax 40X–2500X phase contrast trinocular microscope with an OMAX S35180U3–18Mp camera. Voucher specimens of the peacock mites collected in this study were deposited in the Acarology Collection of the Entomology Laboratory (SL01LA68) at the Universidad Nacional de Piura, Peru. 

### 2.3. The Biometric Characteristics of the Fruits Infested by Peacock Mites 

Fifty of the mamey fruit collected in Section 2.2 were selected to undertake biometric analyses. The biometric characteristics measured are as follows:

− weight of fruit (Wfruit), seeds (Wseed) and epicarp (Wepicarp) (digital scale, precision 0.10 g),
− length of fruit measured using a slide caliper (precision 0.01 mm),
− circumference of fruit determined with a meter ribbon (precision 1 mm).
− weight of pulp (Wpulp) established using the equation,

Wpulp = [Wfruit − (Wepicarp + Wseed)].

− percentage of pulp (P%) using the equation:

$$P\%=\frac{\mathrm{Wpulp}}{\mathrm{Wfruit}}\times 100$$

## 3. Results and Discussion

This is the first record of *Tuckerella pavoniformis* feeding on *Mammea americana* trees (Figure 1, Table 1). This peacock mite is widely distributed throughout the Americas (Table 2), and has been collected from a taxonomically broad range of hosts. It is interesting to note that not only is *T. pavoniformis* restricted to the new world, and is not known from outside the Americas, a large proportion of its hosts plants are endemic to the Americas also (Table 2). Following from this, *T. pavoniformis* is the most widely established tuckerellid species in cocoa producing areas in Piura, Peru, where it occurs in populations large enough to cause significant economic damage to cocoa fruit that results in reduced harvestable yield [25]. The populations of *T. pavoniformis* on mamey reported here are comparably large in size, and further dedicated research on potential yield reduction is warranted.

**Figure 4:**
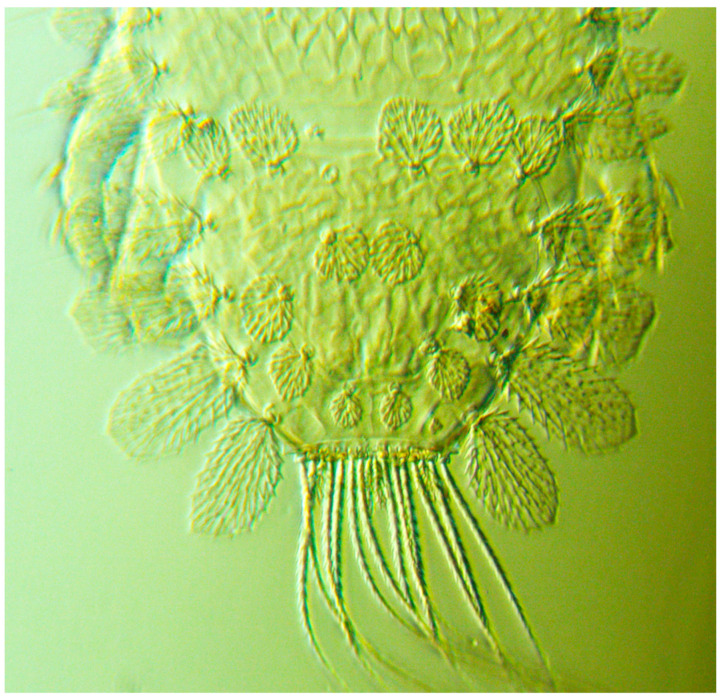
Dorsal view of opisthosoma of adult female *Tuckerella pavoniformis*.

Peacock mites are easily recognized by their outstanding broadly flattened dorsal setae that are usually white in colour and ovate to obovate in shape (i.e., leaf-like in appearance), in conjunction with their striking “tail” setae. *Tuckerella pavoniformis* is separated from other peacock mite species by having six pairs of elongate flagellate caudal setae equal in length (*h2*, *h3*, *h4*, *h5*, *h7*, *h8*) and two pairs of short broad ovate setae (*h1* and *h6*); dorsal cuticle in the pygidial region with mostly regular reticulations; and setae *f2* larger than, and inserted anterior to, setae *f1* (Figure 4).

**Figure 5:**
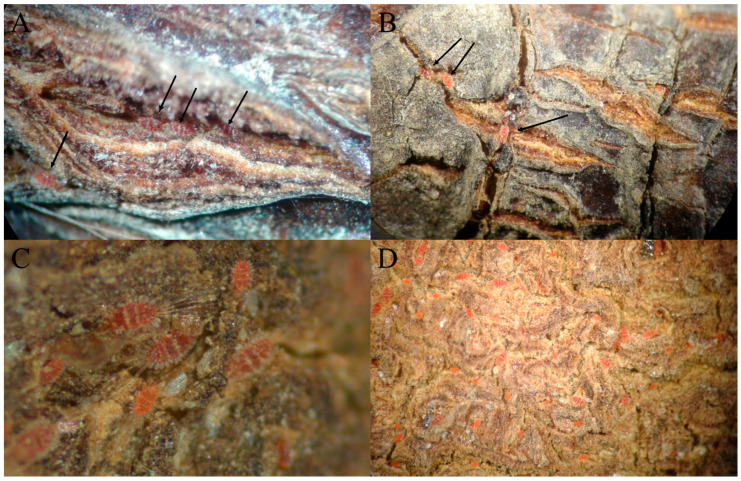
Aggregations of adult *Tuckerella pavoniformis* on *Mammea americana* with one or more eggs and/or immatures (indicated by black arrows) on branchlets sections (**A**,**B**) and fruit epicarp (**C**,**D**).

*Tuckerella pavoniformis* was observed and collected on the branchlets and fruit epicarps of mamey (Figure 5). As aggregations of mites were more common, and the population densities were much higher (mean 10.6 mites/cm^2^), on the fruit than on the branchlets (mean 3.4 mites/15 cm of branchlets) (Table 1), we assume that the fruits represent the preferred feeding site for this species. Motile stages and eggs of *T. pavoniformis* are readily observed wedged within the crevices found on the epicarp of the fruit and on the surface of the branchlets. The mites appear to use these crevices to access feeding and oviposition sites, and for protection from predation and climate. Due to its high respiration rate of 28–40 mg CO_2_ kg^−1^ h^−1^ at 27 °C, mamey is a climacteric fruit and is highly perishable with a limited shelf-life [35,36,37]. As a consequence of this, the fruit will drop from the tree once completely ripened. During this survey, large numbers of mites were observed on fallen ripe fruit that were haphazardly examined in situ with a hand lens. Additionally, incidental observations indicated that peacock mites were also present on the fruit being sold at a local market in Piura, Peru.

The analysis of the biometric characteristics recorded for ripe fruits of *M. americana* infested by *T. pavoniformis* revealed an average weight of 397.23 ± 20.79 g, with individual fruit ranging in weight from 355.40–439.00 g each. In the majority of cases, the fruits of the sampled trees developed only one seed, with average seed number of 1.52 ± 0.11 (Figure 2). The average percentage of pulp per fruit observed was 56.51 ± 1.74% (Table 3). The critical next step is to evaluate the effect that the observed significant populations of peacock mites have on these biometrics, through a comparison with biometrics from healthy fruits, as has been demonstrated previously by Escobar-Garcia et al. [25] for cocoa fruits.

There are few studies regarding the mite diversity on mamey. There are no records of the phytophagous spider mite family Tetranychidae [38] and the predator family Phytoseiidae [39], being collected on *M. americana*. However, De Leon [40] has reported the flat mite “*Brevipalpus phoenicis*” (Geijskes) (Tenuipalpidae) and three species of Tuckerellidae, *T. ornata* and *T. knorri* reported previously by Cao [41], and *T. pavoniformis* reported here from mamey.

*Tuckerella pavoniformis* is an important mite on fruit trees (avocado, cocoa, guava) and ornamental trees, and its presence on *M. americana* in Peru is of potential concern. We consider it to be of phytosanitary importance due to its association with cocoa cultivation in agroforestry systems, as occurs in Singucate, Piura (Figure 1 and Figure 3). In this case, control strategies within Integrated Pest Management programs should be considered, including equipment such as Unmanned Aerial Vehicles (UAV) to assist with efficient management and application of miticides on such tall trees (15 to 20 m). UAVs are highly capable, and their uses are rapidly expanding as they become increasingly adopted across many sectors of agriculture, including for pesticide application [42]. Finally, it is possible that the slow development rate of the fruit may play a significant role in the spread of *T. pavoniformis*. Since the fruit takes 12 months to fully mature, the mites have a long time to build up potentially large populations, after which the fruit is then moved to many parts of Peru for commercialization and consumption. Further research on mite distribution is needed, including sampling mite populations throughout the period of fruit development. Given that these mites often go undetected through concealment on the fruit, we encourage a greater awareness of the domestic movement of this fruit, and the implementation of a post-harvest washing process in an effort to prevent the spread of this mite throughout the rest of the country, before its commercialization is developed further. In addition to preventing or slowing its movement into the main cocoa producing areas where this species is known to cause significant damage to the fruit.

## Figures and Tables

**Table 1:** Locality records for *Tuckerella pavoniformis* in North-western Peru.

Localities/Departament	Collection Date	Coordinates		Average Mites/Branchlets	Average Mites/cm^2^ Fruit Surface
		South	West		
Los Ranchos/Piura	25–Nov.–2020	5°16′50.28″	79°40′16.44″	3.0	17.0
Querecotillo/Piura	03–Dec.–2020	4°50′14.31″	80°39′22.82″	3.5	18.5
Morropón/Piura	03–Dec.–2020	5°19′49.82″	79°52′44.55″	2.0	13.5
Tambogrande/Piura	03–Dec.–2020	4°53′49.50″	80°27′41.40″	3.5	15.5
Tambogrande/Piura	03–Dec.–2020	4°52′51.70″	80°19′19.40″	2.5	16.0
Salitral/Piura	03–Dec.–2020	5°32′21.50″	79°43′10.70″	0.0	4.0
Tambogrande/Piura	04–Dec.–2020	4°54′28.00″	80°20′45.49″	2.0	19.0
Huangala/Piura	04–Dec.–2020	4°50′19.36″	80°36′18.78″	0.0	2.5
Ex CP7 Tambogrande/Piura	04–Dec.–2020	4°54′1.20″	80°15′29.24″	0.0	7.0
Tambogrande/Piura	04–Dec.–2020	4°56′14.48″	80°15′11.50″	0.0	5.0
Morropón/Piura	04–Dec.–2020	5°10′23.28″	79°57′50.40″	2.0	6.0
Tambogrande/Piura	06–Dec.–2020	4°53′35.31″	80°20′32.85″	0.0	13.0
San Juan De Bigote/Piura	06–Dec.–2020	5°17′43.08″	79°42′11.58″	1.5	1.0
Salitral/Piura	06–Dec.–2020	5°30′51.40″	79°42′33.30″	10.5	6.0
Tangarara/Piura	06–Dec.–2020	4°54′0.31″	80°51′8.84″	2.5	5.5
Morropón/Piura	06–Dec.–2020	5°11′34.80″	79°58′11.22″	9.0	8.0
San Juan De Bigote/Piura	06–Dec.–2020	5°19′37.68″	79°43′33.84″	4.0	5.0
La Legua/Piura	06–Dec.–2020	5°14′15.9″	80°39′48.8″	2.0	9.0
La Legua/Piura	06–Dec.–2020	5°14′18.2″	80°39′47.2″	4.0	10.5
Salitral/Piura	07–Dec.–2020	5°19′35.10″	79°52′44.02″	5.0	15.0
Tambogrande/Piura	07–Dec.–2021	4°53′20.72″	80°26′14.85″	0.0	9.5
Tambogrande/Piura	07–Dec.–2022	4°52′20.94″	80°26′11.73″	14.0	23.5
Quemazon/Piura	09–Dec.–2020	5°18′8.83″	79°42′18.55″	7.0	17.5
Singucate/Piura	09–Dec.–2020	5°16′44.44″	79°40′53.59″	4.0	16.0
Buenos Aires/Piura	21–Jan.–2021	5°14′35.63″	79°57′33.25″	8.5	10.0
Los Cedros/Tumbes	11–Oct.–2021	3°36′51.22″	80°31′53.10″	1.5	5.5
Cabuyal/Tumbes	03–Nov.–2021	3°43′6.12″	80°26′7.74″	0.0	8.5

**Table 2:** *Tuckerella pavoniformis* in the Americas—distribution and host plants (hosts in bold are endemic to the Americas).

Location	Host (Family)	References
Brazil	***Malpighia emarginata* D.C. (Malpighiaceae)** *Litchi chinensis* Sonn (Sapindaceae)	[26] [27]
Caribbean	***Annona muricata* L. (Annonaceae)** ***Lantana camara* L. (Verbenaceae)** ***Turnera ulmifolia* L. (Passifloraceae)**	[28] [28] [28]
Costa Rica	***Psidium cattleianum* Sabine (Myrtaceae)** ***Malpighia glabra* L. (Malpighiaceae)** ***Persea americana* Mill. (Lauraceae)**	[6] [29] [30]
Cuba	***Achras sapote* L. (Sapotaceae)** *Casuarina equisetifolia* L. (Casuarinaceae) ***Persea americana***	[31] [31] [32]
Peru	***Theobroma cacao*L. (Malvaceae)** *Cydonia oblonga* Mill. (Rosaceae) ***Mammea americana*** **L. (Calophyllaceae)**	[25] [33] present study
Republica Dominicana	***Persea americana***	[Ochoa, pers.obs.]
USA	*Hibiscus* spp. (Malvaceae) ***Carica papaya* (Caricaceae)** ***Persea americana*** *Quercus* spp. (Fagaceae)	[1,4] [4] [Ochoa, pers.obs.] [34]

**Table 3:** Biometric characteristics of fruits with *T. pavoniformis* populations (n = 50 fruit).

Biometric Characteristics	Means ± SE	95% Confidence Interval
Average fruit weight (g)	397.23 ± 20.79	355.40–439.00
Average pulp weight (g)	228.28 ± 15.21	197.70–258.85
Average seed weight (g)	87.34 ± 5.36	76.57–98.10
Average fruit height (cm)	9.02 ± 0.13	8.74–9.28
Average fruit circumference (cm)	28.96 ± 0.52	27.90–30.00
Average seed number	1.52 ± 0.11	1.30–1.73
Percentage of pulp (%)	56.51 ± 1.74	53.02–60.00
Percentage of seed (%)	22.29 ± 0.95	20.37–24.20
Percentage of epicarp (%)	21.2 ± 1.03	19.12–23.26
Ratio pulp/seed	2.88 ± 0.17	2.52–3.22

## Data Availability

Not applicable.
